# Investigating the Effects of Surface Texture Direction and Poisson’s Ratio on Stress Concentration Factors

**DOI:** 10.3390/ma18102260

**Published:** 2025-05-13

**Authors:** Yuwei Liu, Yuhui Liu, Zhengkun Cheng

**Affiliations:** 1 School of Mechanical and Electrical Engineering, China University of Mining and Technology-Beijing, Beijing 100083, China; yuweiliu1@126.com (Y.L.); liuyh6161@163.com (Y.L.); 2 School of Undergraduate Education, Shenzhen Polytechnic University, Shenzhen 518055, China

**Keywords:** stress concentration factor, analytical solution, Poisson’s ratio, surface texture direction, finite element analysis

## Abstract

A three-dimensional (3D) analytical framework was developed to quantify stress concentration factors (SCFs) on engineering surfaces with arbitrary slight undulations, effectively addressing the limitations of existing two-dimensional (2D) models by rigorously integrating the effects of Poisson’s ratio (v) and surface texture directionality (θ). Initially, the 3D analytical solutions for single-notched specimens under uniaxial loading, which account for the v effect, were derived and compared with their 2D counterparts. The results demonstrate a clear positive correlation between v and SCFs. Subsequently, the framework was extended to single-layer undulating surfaces, revealing anisotropic stress modulation governed by θ. SCFs increase monotonically with θ, a directional sensitivity that 2D solutions are unable to represent. A parametric analysis of cosine-wave surfaces further identified a nonlinear accuracy dependency on the amplitude–frequency product (Af). Finite element method (FEM) validation showed that the relative errors are less than 5% when Af<0.05, and they rise to 14.8% when Af≈0.1. Furthermore, application to machined surfaces validated the superior accuracy of the 3D solution, achieving approximately 10% improvement compared to 2D methods with errors controlled within 5%. Significantly, the texture direction perpendicular to the loading direction results in notably higher SCFs than the parallel direction, directly correlating texture orientation with stress concentration severity. This study provides a robust theoretical basis for surface topography optimization in engineering applications, with validated reliability across geometric and material parameters.

## 1. Introduction

The influence of surface defects on the stress concentration of materials holds significant research value in the fields of structural design and fatigue analysis [1]. Surface defects not only lead to a notable increase in local stress but also often serve as initiation points for crack nucleation and propagation, thereby severely affecting the mechanical properties and service life of materials [2,3]. Studies have shown that the morphological characteristics [4], geometric dimensions [5], and spatial distribution [6] of defects exhibit significant correlations with the degree of stress concentration, and these correlations vary markedly depending on the material type. Specifically, in metallic materials, surface cracks and voids often induce pronounced stress concentrations, thereby accelerating the initiation of fatigue cracks [7,8]. For composite materials, the influence mechanism of surface defects on stress distribution is more complex, with extensive research confirming that the geometric shape and spatial location of defects play a decisive role in the distribution characteristics of the stress field [9]. Notably, with the rapid development of additive manufacturing (AM) technology, the impact of surface defects inherent to this process on the stress concentration behavior is becoming increasingly prominent [10]. Research studies, including both the traditional methods [11,12,13] and the machine learning approaches [14,15,16], indicate that porosity defects [17,18], surface irregularities [19], and microstructural inhomogeneity [20] generated during the AM process can significantly reduce the static strength and fatigue performance of the components [21,22]. These investigations confirm that optimizing the AM process parameters to control surface defects can significantly reduce stress concentration levels, thereby enhancing the fatigue performance of materials [23,24]. In this study, the Al-Mg-Sc high-strength aluminum alloy—a material system particularly well suited for laser-based AM processes, owing to its superior processability and age-hardening potential—was fabricated using Laser Melting Deposition (LMD) technology. The as-built specimens exhibited characteristic surface roughness, which is inherent to the powder bed fusion process, along with sub-surface defects such as partially melted particles and gas-entrapped pores. Subsequent grinding processes were utilized to produce controlled surface textures featuring directional machining marks, thereby establishing a comparative framework for investigating both the as-printed and post-processed surface conditions.

In terms of the research methodologies in this field, traditional techniques including empirical formulae [11,25,26,27], FEM simulations [12,28,29,30], and experimental validation [13,31], as well as the contemporary deep learning algorithms [14,15,16,32], are widely employed. In engineering applications, empirical formulae assume surface topography as successive adjacent notches. The roughness height parameters, in terms of the average roughness ($R_{a}$), peak-to-valley height roughness ($R_{y}$), 10-point roughness ($R_{z}$), and the valley effective root radius ($\bar{\rho }$), are incorporated into the prediction formulae, and the representative prediction formulae include the Neuber rule [33] and the Arola–Ramulu model [34]. Gu et al. extended the Arola–Ramulu model by integrating surface skewness and kurtosis parameters [35]. Empirical models relying on roughness parameters fail to capture the distribution characteristic of stress concentration along the machined surface profile. Moreover, existing empirical models predominantly rely on the 2D roughness parameters, while 3D parameters such as the arithmetic mean height parameter $S_{a}$ are scarcely taken into account in these prediction models. Numerical simulations and experimental investigations have become primary approaches for studying the influence of surface defects on stress concentration. Through combined FEM analysis and experimental testing, researchers can accurately predict the stress concentration behavior and crack initiation sites induced by defects, providing reliable theoretical foundations for fatigue life prediction [36,37]. However, the FEM simulations are confronted with the dilemma of computational efficiency, as each measured surface topography requires the establishment of a separate multi-scale finite element model [38,39]. Additionally, deep learning algorithms offer a new perspective for revealing the relationship between surface roughness and SCFs [14,15,16,32]; however, this approach requires a substantial amount of high-quality datasets for training to ensure prediction accuracy.

By comparison, analytical solutions present an attractive alternative for predicting the SCFs induced by surface roughness [40,41,42,43,44], which is not only capable of elucidating the fundamental physical mechanisms but also offers significant advantages in terms of computational efficiency [45]. For 2D surface profiles, Gao developed an analytical solution for SCFs in sinusoidal shallow surfaces [40]. Subsequently, Medina established an SCF-formula-generating equation for arbitrary, 2D, slightly undulating surfaces [46]. Building on these foundations, Cheng extended the research to real machined surfaces based on Fourier representation [42,47]. Expanding the scope of this research, Cheng et al. addressed stress concentration in 3D micro-undulating surfaces under uniaxial/biaxial boundary loading conditions, and they developed an analytical solution applicable to 3D real machined surface topographies [44]. Notably, the relationship between the analytical solutions for SCFs of 2D surface profiles and those of 3D surface topographies has not yet been thoroughly discussed. In engineering applications, whether the 2D SCFs can replace the 3D SCFs is a debatable issue, as the 2D simplification does not encompass the material’s Poisson’s ratio and surface texture information. Scholars have demonstrated that the surface texture direction significantly affects stress concentration behavior and reduces the fatigue performance of specimens [48,49]. The impetus for this study is to elucidate the underlying mechanisms by which the material’s Poisson’s ratio and surface texture characteristics affect the SCFs, from the perspective of analytical solutions.

This paper is organized as follows: First, a general analytical solution for SCFs in 3D micro-undulating surfaces was established based on the boundary perturbation method. Subsequently, the solution was applied to single-notched geometric surfaces under uniaxial tensile loading, with a detailed analysis of the influence of Poisson’s ratio v and the notch geometric configuration on the maximum SCFs. Furthermore, the theoretical model was extended to the case of single-layer cosine surfaces, where systematic theoretical analysis and numerical simulations revealed the effects of Poisson’s ratio v and the surface texture direction angle θ on SCFs. Additionally, the theoretical solutions were validated by FEM results, and the nonlinear influence of the limiting condition Af≪1 on the accuracy of the analytical solution was also revealed. Finally, by comparing the FEM results of the real machined surface topography measured from the additively manufactured specimen with the theoretical solution, the influence of v and θ on the stress concentration behavior of actual engineering surfaces was thoroughly investigated.

## 2. General Analytical Solutions for SCFs

**Figure 1:**
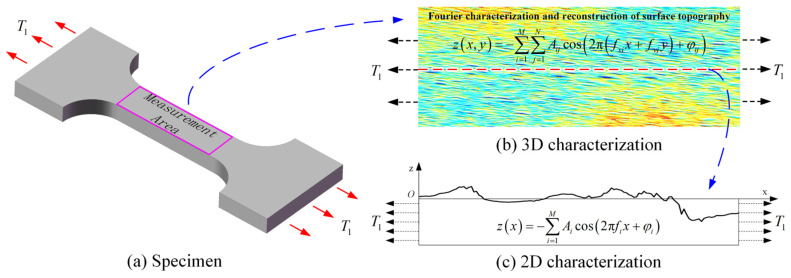
Schematic of the specimen and surface topography characterization.

In engineering analysis, 2D contour or 3D topography measurements are typically performed on critical regions of components to obtain 2D/3D characterization parameters and geometric features of surface morphology. These data are further used to evaluate their effects on stress concentration problems [17,24]. This study focuses on the effect of surface morphology on SCFs under uniaxial tensile loading, with the test specimen illustrated in Figure 1. The central region of the specimen subjected to uniaxial tensile load $T_{1}$ was selected as the measurement area. The 3D surface morphology was measured and characterized, the centerline profile was extracted, and a Fourier representation method was applied to the measured surface morphology to investigate its effect on SCFs [44,47].

**Figure 2:**
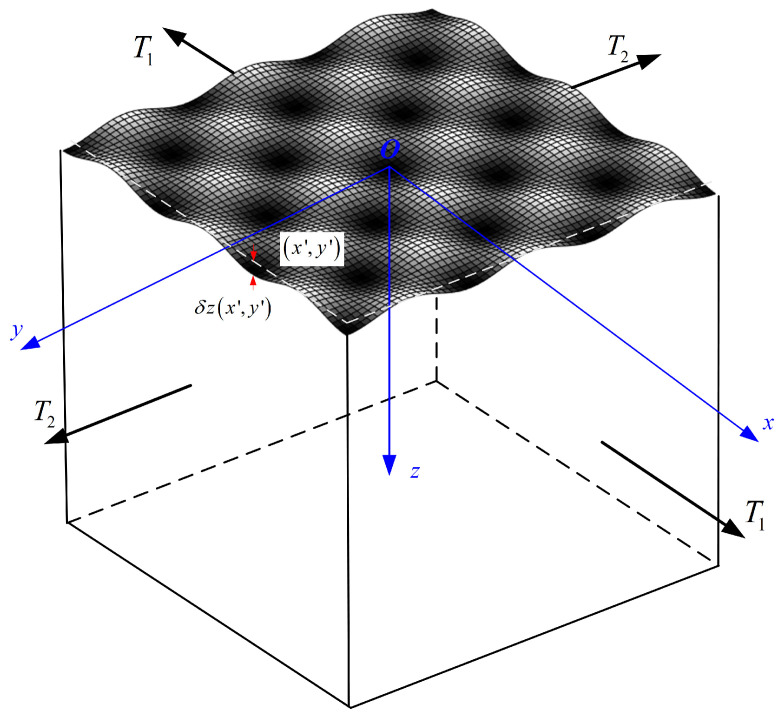
Surface topography with a perturbation $\delta z\left(x^{\prime },y^{\prime }\right)$ at each point $\left(x^{\prime },y^{\prime }\right)$ along the x−y plane.

The 3D geometry of the elastic semi-infinite space was subjected to tensional stresses with $\sigma_{xx}^{0}=T_{1}$ and $\sigma_{yy}^{0}=T_{2}$, and the loading ratio was $C=T_{2}/T_{1}$, as shown in Figure 2. A rough surface morphology can be conceptualized as an ideal smooth surface subjected to a microscale perturbation $\delta z\left(x^{\prime },y^{\prime }\right)$ at each point $\left(x^{\prime },y^{\prime }\right)$.

Cheng et al. derived the surface displacements and stresses for arbitrary micro-perturbed surface topographies using a first-order boundary perturbation approach [44]. The displacements and stresses along the rough surface can be expressed as the sum of the reference values and the perturbed terms:

(1) $$\begin{array}{c} u_{i}\left(x,y,z\right)=u_{i}^{0}\left(x,y,z\right)+\delta u_{i}\left(x,y,z\right),\\ \sigma_{ij}\left(x,y,z\right)=\sigma_{ij}^{0}\left(x,y,z\right)+\delta \sigma_{ij}\left(x,y,z\right), \end{array}$$

where $u_{i}\left(x,y,z\right)$ denotes the displacements, $\sigma_{ij}\left(x,y,z\right)$ represents the stress, $u_{i}^{0}\left(x,y,z\right)$ and $\sigma_{ij}^{0}\left(x,y,z\right)$ are the reference values of displacement and stress, respectively, and $\delta u_{i}\left(x,y,z\right)$ and $\delta \sigma_{ij}\left(x,y,z\right)$ correspond to the perturbation terms for displacement and stress, respectively.

By relocating the reference plane to a distance $z\left(x,y\right)$ from the x−y plane, the surface stress components of the 3D micro-perturbed surface can be expressed as follows [44]:

(2) $$\sigma_{xx}\left(x,y\right)=T_{1}-\int_{-\infty }^{+\infty }\int_{-\infty }^{+\infty }\overset{\wedge }{G_{xx}}\left[z\left(x^{\prime },y^{\prime }\right)-z\left(x,y\right)\right]dx^{\prime }dy^{\prime },$$

(3) $$\sigma_{yy}\left(x,y\right)=T_{2}-\int_{-\infty }^{+\infty }\int_{-\infty }^{+\infty }\overset{\wedge }{G_{yy}}\left[z\left(x^{\prime },y^{\prime }\right)-z\left(x,y\right)\right]dx^{\prime }dy^{\prime },$$

(4) $$\sigma_{xy}\left(x,y\right)=-\int_{-\infty }^{+\infty }\int_{-\infty }^{+\infty }\overset{\wedge }{G_{xy}}\left[z\left(x^{\prime },y^{\prime }\right)-z\left(x,y\right)\right]dx^{\prime }dy^{\prime }.$$

If $z\left(x,y\right)$ is independent of y, Equations (2)–(4) can be simplified into their 2D counterparts. These equations define the analytical solutions for the stress distribution in 3D undulating surfaces subjected to tensile or pure bending conditions, where the kernel functions are given as follows [44]:

(5) $$\begin{array}{c} \overset{\wedge }{G_{xx}}=\frac{T_{1}}{\pi s^{7}}\left[\left(3vC-2v-2\right){\left(x^{\prime }-x\right)}^{4}+\left(1-2v\right){\left(y^{\prime }-y\right)}^{4}+\left(11v-12vC-1\right){\left(x^{\prime }-x\right)}^{2}{\left(y^{\prime }-y\right)}^{2}\right],\\ \overset{\wedge }{G_{yy}}=\frac{T_{1}}{\pi s^{7}}\left[C\left(1-2v\right){\left(x^{\prime }-x\right)}^{4}+\left(3v-2vC-2C\right){\left(y^{\prime }-y\right)}^{4}+\left(11vC-12v-C\right){\left(x^{\prime }-x\right)}^{2}{\left(y^{\prime }-y\right)}^{2}\right],\\ \overset{\wedge }{G_{xy}}=\frac{T_{1}}{\pi s^{7}}\left[\left(18vC-12v-3C-3\right){\left(x^{\prime }-x\right)}^{3}\left(y^{\prime }-y\right)+\left(18v-12vC-3C-3\right)\left(x^{\prime }-x\right){\left(y^{\prime }-y\right)}^{3}\right], \end{array}$$

where v is the Poisson’s ratio and $s=\sqrt{{\left(x^{\prime }-x\right)}^{2}+{\left(y^{\prime }-y\right)}^{2}}$.

In engineering applications, SCFs can be defined as the ratio of the actual stress in the perturbed region to the reference stress. In this study, the boundary loads $T_{1}$ and $T_{2}$ were adopted as reference stresses, and the SCFs are given by

(6) $$\begin{array}{c} K_{t}^{T_{1}}\left(x,y\right)=\sigma_{xx}\left(x,y\right)/T_{1},\\ K_{t}^{T_{2}}\left(x,y\right)=\sigma_{yy}\left(x,y\right)/T_{2},\\ \;K_{ts}\left(x,y\right)=\sigma_{xy}\left(x,y\right)/\left(T_{1}+T_{2}\right), \end{array}$$

where $K_{t}^{T_{1}}$ and $K_{t}^{T_{2}}$ are the normal SCFs along the $T_{1}$ and $T_{2}$ directions, respectively, and $K_{ts}$ denotes the shear SCFs.

## 3. Analytical Solutions for Representative Geometric Surfaces

Surface defects such as pitting, dents, scratches, and surface roughness usually introduce stress concentration, thereby affecting the service life and reliability of the components [22]. In response to this issue, this section systematically investigates the influence mechanisms of single-notch geometric surfaces and single-layer cosine-wave surfaces on stress concentration.

Under uniaxial tension/compression loading conditions (the loading ratio $C=T_{2}/T_{1}=0$), Equation (6) is applied to semi-ellipse single-notch, sinusoidal single-notch, Gaussian notch, and single-layer cosine-wave surfaces. The surface morphology $z\left(x,y\right)$ constitutes a real continuous function satisfying the Hölder condition within its domain [44], where the slope for each surface point needs to be of a sufficiently small magnitude. It should be noted that the shallow condition of the single-notch geometric surfaces requires that the depth-to-width ratio be far less than 1, while the limitations for a single-layer cosine-wave surface require that the amplitude–frequency product be far less than 1.

### 3.1. Single-Notch Geometric Surfaces

**Figure 3:**
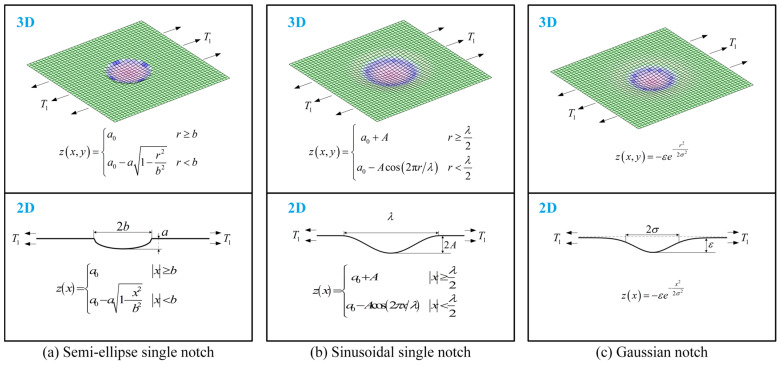
Different types of single notches under uniaxial tensile loading.

Figure 3 illustrates three representative single-notch geometric configurations: semi-elliptical, sinusoidal, and Gaussian notch.

(1) A semi-ellipse single notch

The equation for the 3D surface is as follows:

(7) $$z\left(x,y\right)=\left\{\begin{array}{cc} a_{0} & r\geq b\\ a_{0}-a\sqrt{1-\frac{r^{2}}{b^{2}}} & r<b, \end{array}\right.$$

where $r=\sqrt{x^{2}+y^{2}}$, while a and b represent the notch depth and half-width, respectively, with a≪2b.

It is evident that the surface stress reaches its maximum value at r=0. Substituting Equation (7) into Equation (6) reveals that the shear SCF $K_{ts}$ equals zero, while the normal SCF is given by

(8) $$K_{t}^{T_{1}}\left(r=0\right)=1+\frac{\pi }{8}\left(v+4\right)\frac{a}{b}.$$

The equation for the 2D semi-elliptical notch is as follows:

(9) $$z\left(x\right)=\left\{\begin{array}{cc} a_{0} & \left|x\right|\geq b\\ a_{0}-a\sqrt{1-\frac{x^{2}}{b^{2}}} & \left|x\right|<b, \end{array}\right.$$

and the SCF at the valley of the notch is shown below [46]:

(10) $$K_{t}\left(x=0\right)=1+2\frac{a}{b}.$$

(2) A sinusoidal single notch

The equation for the 3D surface is as follows:

(11) $$z\left(x,y\right)=\left\{\begin{array}{cc} a_{0}+A & r\geq \frac{\lambda }{2}\\ a_{0}-A\cos\left(2\pi r/\lambda \right) & r<\frac{\lambda }{2}, \end{array}\right.$$

where A and λ denote the amplitude and wavelength of the sinusoidal notch, respectively, with 2A≪λ. The surface stress reaches its maximum at r=0, similar to the semi-elliptical notch case, where the shear stress is zero. The normal SCF is expressed as follows:

(12) $$K_{t}^{T_{1}}\left(r=0\right)=1+2.90902\left(v+4\right)A/\lambda .$$

The 2D sinusoidal notch and its maximum SCF are presented below [46]:

(13) $$z\left(x\right)=\left\{\begin{array}{cc} a_{0}+A & \left|x\right|\geq \frac{\lambda }{2}\\ a_{0}-A\cos\left(2\pi x/\lambda \right) & \left|x\right|<\frac{\lambda }{2}, \end{array}\right.$$

(14) $$K_{t}\left(x=0\right)=1+4\pi \frac{A}{\lambda }.$$

(3) A Gaussian notch

The equation for the 3D surface is as follows:

(15) $$z\left(x,y\right)=-\varepsilon e^{-\frac{r^{2}}{2\sigma^{2}}},$$

where σ is the standard deviation, the width parameter is 2σ, and ε is the depth parameter ensuring the shallow-layer condition, with ε≪2σ. The surface stress attains its maximum at r=0, where the shear stress is zero. The normal SCF is expressed as follows:

(16) $$K_{t}^{T_{1}}\left(r=0\right)=1+\frac{\sqrt{2\pi }\varepsilon }{8\sigma }\left(v+4\right).$$

The 2D Gaussian curve and its maximum SCF are presented below:

(17) $$z\left(x\right)=-\varepsilon e^{-\frac{x^{2}}{2\sigma^{2}}},$$

(18) $$K_{t}(x=0)=1+\frac{2\sqrt{2}\varepsilon }{\sqrt{\pi }\sigma }.$$

From the formulae of the 2D and 3D SCFs for each single notch, the ratio R of the maximum stress perturbation component can be obtained as follows:

(19) $$R=\frac{K_{t}^{T_{1}}-1}{K_{t}-1}.$$

**Figure 4:**
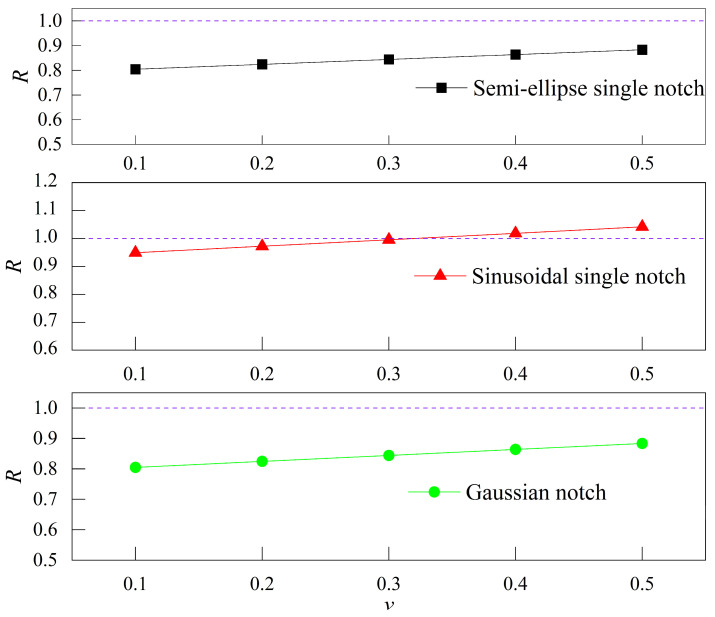
Relationship between the maximum stress perturbation component ratio R and Poisson’s ratio v.

When R<1, it indicates that, in guiding product design, the 2D analytical solution is more conservative compared to the 3D counterpart; when R>1, the 2D analytical solution becomes relatively more aggressive. The relationship between the maximum stress perturbation component ratio R and Poisson’s ratio v is shown in Figure 4.

As shown in Figure 4, when the v ranges from 0.1 to 0.5, the maximum stress perturbation component ratio R for both the semi-ellipse single notch and the Gaussian notch is less than 1, which indicates that the 2D analytical solution is relatively conservative. For the sinusoidal single notch, the 2D analytical solutions are relatively conservative with v= 0.1, 0.2, or 0.3; conversely, they are relatively aggressive when v= 0.4 or 0.5.

**Figure 5:**
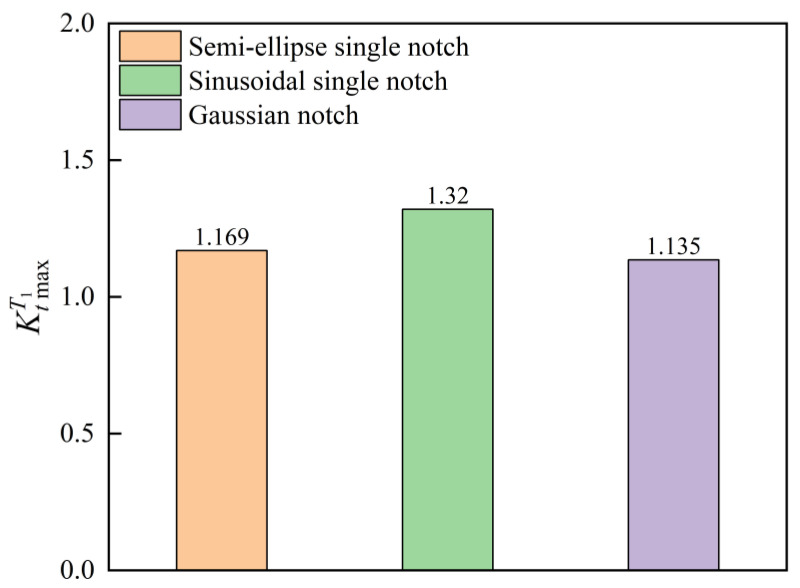
The 3D SCFs for different single-notch geometries with identical notch depth, width, and Poisson’s ratio.

When the notch depth a=2A=ε=0.01 mm, width λ=2b=2σ=0.2 mm, and v=0.3 for the three types of single notch surfaces, the analytical solutions of 3D SCFs for each type of single notch are calculated as shown in Figure 5.

Figure 5 demonstrates that the influence of notch shape on the SCFs is significant and cannot be ignored. While some scholars simplify surface notches to semi-ellipse configurations and employ Neuber’s model for SCF prediction [33], such simplification is not rigorous enough.

### 3.2. Single-Layer Cosine-Wave Surfaces

Single-layer cosine-wave surfaces can be categorized into two types: orthogonal and non-orthogonal wave surfaces. Orthogonal wave surfaces refer to those where the fluctuations in different directions are independent and orthogonal to each other, thus lacking information on the texture direction. In contrast, non-orthogonal wave surfaces possess directional characteristics with explicitly defined texture direction information.

While Gao et al. established analytical solutions for SCFs in orthogonal 3D surfaces under biaxial tensile loading [40], this section derives the SCF analytical solution for orthogonal surfaces subjected to uniaxial tensile loading. The formula for the orthogonal 3D cosine-wave surface is given by

(20) $$z\left(x,y\right)=-A\cos\left(2\pi \frac{x}{\lambda }\right)\cos\left(2\pi \frac{y}{\lambda }\right),$$

**Figure 6:**
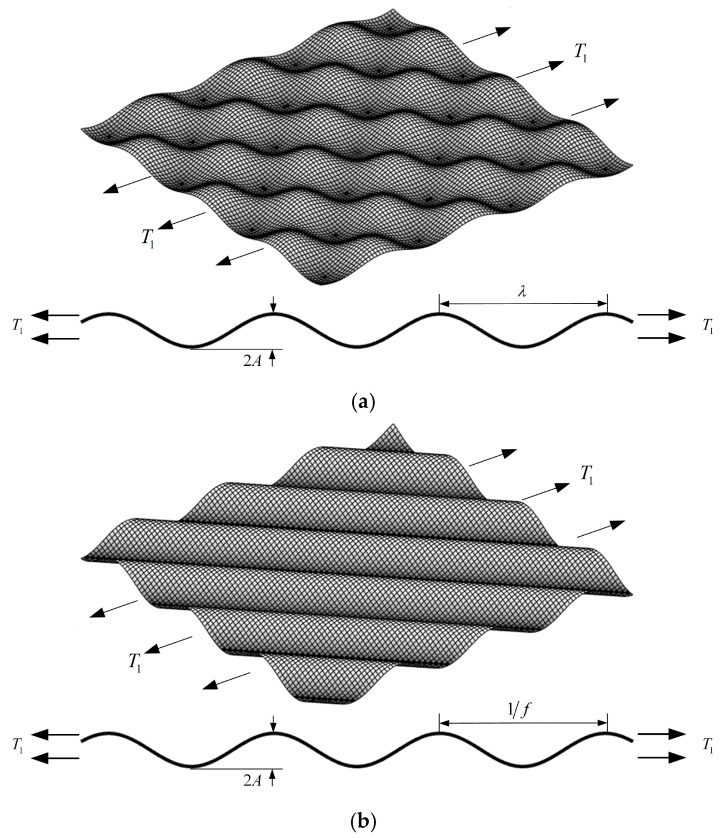
Orthogonal and non-orthogonal cosine-wave surfaces under uniaxial tensile loading: (**a**) Orthogonal cosine-wave surface. (**b**) Non-orthogonal cosine-wave surface.

As shown in Figure 6a, where A denotes the amplitude and λ represents the surface wavelength, the 3D SCFs for the orthogonal wave surface under uniaxial tensile loading become

(21) $$K_{t}^{T_{1}}\left(x,y\right)=1+\sqrt{2}\pi \left(2+v\right)\frac{A}{\lambda }\cos\left(2\pi \frac{x}{\lambda }\right)\cos\left(2\pi \frac{y}{\lambda }\right).$$

For the orthogonal cosine-wave surfaces, the maximum stress concentration occurs at the wave troughs, where the maximum value of the 3D SCF becomes

(22) $$K_{t\max}^{T_{1}}=1+\sqrt{2}\pi \left(2+v\right)\left(A/\lambda \right).$$

Cheng et al. derived the analytical solutions for the SCFs of non-orthogonal 3D surfaces [44]. For the non-orthogonal 3D single-layer cosine surfaces, the frequencies in the x and y directions are $f_{x}$ and $f_{y}$, respectively, with an amplitude of A, and the constraint condition is Af≪1, as shown in Figure 6b. The formula for this 3D surface is given as follows:

(23) $$z\left(x,y\right)=-A\cos\left(2\pi (f_{x}x+f_{y}y)\right).$$

The texture direction parameter θ of the single-layer wave surface is determined by the surface frequencies $f_{x}$ and $f_{y}$, and the relationship is expressed as follows:

(24) $$\theta =\mathrm{arccot}\frac{f_{y}}{f_{x}}\left(f_{x}\neq 0\right).$$

Substituting Equation (24) into Equation (23) yields the 3D surface expression (Equation (25)), with the surface texture direction parameter θ. When C=0, substituting Equation (25) into Equation (6) gives the 3D SCF formula of the non-orthogonal cosine-wave surface (Equation (26)).

(25) $$z\left(x,y\right)=-A\cos\left(2\pi f_{x}\left(x+y\cot\theta \right)\right),$$

(26) $$K_{t}^{T_{1}}\left(x,y\right)=1+4\pi Af_{x}\frac{1+\left(1+v\right)\cot^{2}\theta }{\csc^{3}\theta }\cos\left(2\pi f_{x}\left(x+y\cot\theta \right)\right)\;.$$

**Figure 7:**
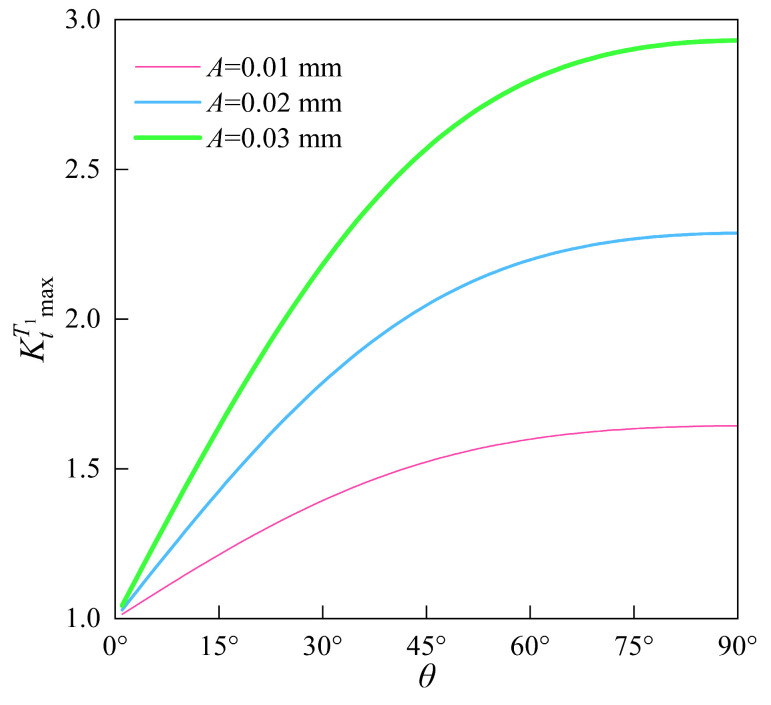
The influence of the texture direction parameter θ on the maximum normal SCF ${K_{t}^{T_{1}}}_{\max}$ for non-orthogonal cosine-wave surfaces under uniaxial tensile loading.

When the amplitude A is 0.01 mm, 0.02 mm, or 0.03 mm, with $f_{x}=5.12{\;\mathrm{mm}}^{-1}$ and v=0.3, the relationships between the maximum normal SCF ${K_{t}^{T_{1}}}_{\max}$ and the texture direction parameter θ are as shown in Figure 7.

As shown in Figure 7, the maximum SCF ${K_{t}^{T_{1}}}_{\max}$ for the non-orthogonal cosine-wave surfaces increases with the increase in the texture direction parameter θ. When θ approaches 0°, ${K_{t}^{T_{1}}}_{\max}$ asymptotically tends to 1, and when $\theta =90^{\circ }$, ${K_{t}^{T_{1}}}_{\max}$ reaches its maximum value. This is consistent with the research pattern reported by Toyoda et al. [48].

When the orthogonal and non-orthogonal cosine-wave surfaces share identical amplitude A and frequency f=1/λ (for example, with v=0.3, A=0.01 mm, and $f=1/\lambda =5.12{\;\mathrm{mm}}^{-1}$), the orthogonal cosine-wave surface with no texture direction information exhibits a constant maximum SCF of 1.523 according to Equation (21). However, the maximum SCF for the non-orthogonal cosine-wave surface, calculated using Equation (26), is not a constant value. As shown in Figure 7, its maximum value increases with the increase n the texture direction parameter θ.

When $f_{x}=f_{y}=f$ and C=0—that is, under uniaxial tensile loading with $\theta =45^{\circ }$—the 3D SCF for the surface is

(27) $$K_{t}^{T_{1}}\left(x,y\right)=1+4\pi Af\beta \cos\left(2\pi f\left(x+y\right)\right),$$

where the coefficient $\beta =\frac{2+v}{2\sqrt{2}}$, which is influenced by the Poisson’s ratio v. It can be observed that the coefficient β increases linearly with the increase in v.

For the SCFs of a 2D cosine wave, assuming A is the amplitude and f is the frequency, with the constraint Af≪1, the surface profile formula is as follows:

(28) $$z\left(x\right)=-A\cos\left(2\pi f\cdot x\right).$$

Therefore, the SCF at each point along the 2D cosine-wave surface profile is as follows [47]:

(29) $$K_{t}\left(x\right)=1+4\pi Af\cos\left(2\pi f\cdot x\right),$$

the maximum SCF occurs at the trough of the cosine-wave profile, and it is given by $K_{t\max}=1+4\pi Af$.

By comparing Equations (27) and (29), it is evident that the coefficient β becomes less than 1 due to the inclusion of Poisson’s ratio v, resulting in a lower maximum value for the 3D analytical solution compared to its 2D counterpart. Consequently, using the 2D analytical solution to evaluate the SCFs is relatively conservative.

In engineering applications, 3D surface stress concentration analysis is often simplified into a 2D extraction method based on a single profile line [43]. Although this simplified approach demonstrates distinct advantages in computational efficiency, it inevitably overlooks the potential impacts of the integrity characteristics of 3D surface morphology on SCFs, particularly the mechanistic roles of critical parameters such as Poisson’s ratio v and the texture direction parameter θ. From a theoretical analysis perspective, in the 3D SCF formula (Equation (27)), v and θ serve as significant variables that markedly affect the distribution characteristics of SCFs. In contrast, traditional 2D simplified formulae (Equation (29)) do not account for these critical parameters, resulting in substantial limitations in both prediction accuracy and application scope.

## 4. Finite Element Validation

This section employs a comparative analysis method to systematically examine the theoretical discrepancies between 3D and 2D analytical solutions, focusing on the influence patterns of v (Poisson’s ratio) and θ (texture direction parameter) on surface stress concentration effects. Further analysis explores the impact of the constraint condition Af on the accuracy of analytical solutions. Concurrently, to validate the effectiveness of the analytical solutions, finite element models (FEMs) of specimens with perturbed surfaces are established. The analytical results provide a more reliable theoretical foundation for engineering practices.

### 4.1. Establishment of FEM

**Figure 8:**
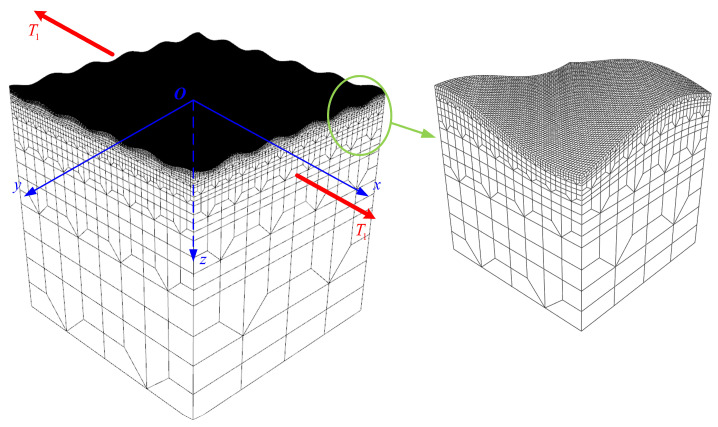
Three-dimensional FEM of single-layer cosine-wave surface morphology.

To validate the influence patterns of single-layer cosine-type 3D surface morphologies on SCFs and the accuracy of theoretical predictions, this study developed an FEM based on the continuum mechanics framework and the assumption of isotropic homogeneous materials. The geometric model was initially meshed using the ABAQUS 2022 finite element software, followed by the application of a custom MATLAB R2022b script to iteratively adjust the surface nodal coordinates based on predefined cosine-wave functions, thereby precisely reconstructing the target 3D surface morphologies. The model employed a cubic geometric configuration with 1 mm edge length, where the top surface was modified by nodal adjustments to generate 3D surface morphologies with cosine-wave features. For numerical simulations, quadratic hexahedral elements were employed for model discretization, with a local mesh refinement strategy implemented in surface morphology regions, ensuring numerical convergence of stress distributions. The element size in the surface morphology region was controlled within 2 μm, satisfying the precision requirements of numerical simulation. A uniform tensile load, $T_{1}=1\;\mathrm{MPa}$, was applied on both sides along the x-axis (as shown in Figure 8). The integration of ABAQUS meshing capabilities and MATLAB-based nodal coordinate manipulation ensured both geometric fidelity and computational efficiency. This hybrid computational workflow leverages the strengths of both platforms, combining ABAQUS’s robust meshing algorithms with MATLAB’s flexibility in handling complex surface parametrization, to achieve high-precision modeling of textured surfaces.

### 4.2. The Influence of Surface Texture Direction on SCFs

Based on Equation (23), three single-layer cosine-type 3D surfaces with a side length of 1 mm were constructed, featuring the following geometric parameters: surface amplitude A=0.01 mm, surface frequency $f=5.12{\;\mathrm{mm}}^{-1}$, and surface texture direction parameters θ= 0°, 45°, and 90°, respectively. The expressions for the 3D surface morphologies were formulated as follows:

(30) $$\begin{array}{llll} Surface & \;I\;: & z\left(x,y\right)=0.01\cos\left(2\pi \cdot 5.12\cdot y\right) & \left(\theta =0^{\circ }\right)\\ Surface & \mathrm{II}: & z\left(x,y\right)=0.01\cos\left[2\pi \cdot 5.12\cdot \left(x+y\right)\right] & \left(\theta =45^{\circ }\right)\\ Surface & \mathrm{III}: & z\left(x,y\right)=0.01\cos\left(2\pi \cdot 5.12\cdot x\right) & \left(\theta =90^{\circ }\right). \end{array}$$

By setting y=0.5 mm in Equation (30), the expressions for the centerline profile curve of the 3D surface can be obtained as follows:

(31) $$\begin{array}{cc} Centerline & \;I\;:\;z\left(x\right)=0.01\cos\left(5.12\pi \right)\\ Centerline & \;\mathrm{II}:z\left(x\right)=0.01\cos\left[2\pi \cdot 5.12\cdot \left(x+0.5\right)\right]\\ Centerline & \mathrm{III}:z\left(x\right)=0.01\cos\left(2\pi \cdot 5.12\cdot x\right). \end{array}$$

**Figure 9:**
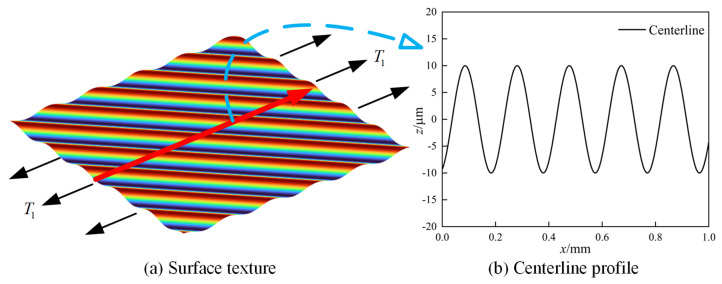
Surface morphology and centerline profile of Surface II.

Taking Surface II as an example, the surface morphology and centerline profile are illustrated in Figure 9. Figure 9a displays the 3D surface morphology and its boundary condition, while Figure 9b presents the surface centerline profile.

Assuming the material v=0.3, the analytical solution $K_{t}^{T_{1}}$ for the 3D surface-normal SCFs can be calculated using Equation (26). The numerical solution $K_{t\left|FEM\right.}^{T_{1}}$ for the surface-normal SCFs is obtained through finite element analysis, while the 2D analytical solution $K_{t}$ for the SCFs at the surface centerline is determined via Equation (29).

**Figure 10:**
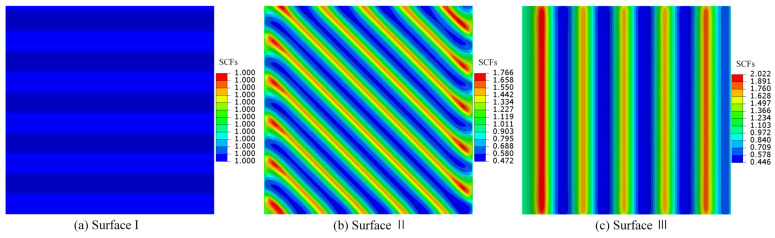
FEM simulation for single-layer cosine-wave surfaces with θ = 0°, 45°, and 90°.

The FEM simulation results for single-layer cosine-type surface morphologies with surface texture direction parameters θ= 0°, 45°, and 90° are presented in Figure 10. It can be observed that the maximum SCFs ${K_{t\left|FEM\right.}^{T_{1}}}_{\max}$ from the simulation results increase with the texture direction parameter θ. When $\theta =0^{\circ }$, $K_{t}$, $K_{t}^{T_{1}}$, and $K_{t\left|FEM\right.}^{T_{1}}$ are all equal to 1; when $\theta =90^{\circ }$, $K_{t}^{T_{1}}$ and $K_{t\left|FEM\right.}^{T_{1}}$ reach their maximum values. However, for $\theta \neq 0^{\circ }$, $K_{t}$ remains constant at 1.643.

Table 1 presents a comparison between the maximum FEM simulation values ${K_{t\left|FEM\right.}^{T_{1}}}_{\max}$ and the maximum 3D analytical solution values $K_{t\max}^{T_{1}}$ for SCFs in different cosine-wave surfaces at locations far from the loading surface. The parameter Af represents the constraint condition for the analytical solution formula of SCFs.

As evidenced by the data in Table 1, it can be observed that, when θ is constant, both of the maximum SCFs (${K_{t\left|FEM\right.}^{T_{1}}}_{\max}$ and $K_{t\max}^{T_{1}}$) exhibit a positive correlation with the parameter Af. Correspondingly, when Af remains constant, both ${K_{t\left|FEM\right.}^{T_{1}}}_{\max}$ and $K_{t\max}^{T_{1}}$ demonstrate a monotonically increasing trend with rising θ.

**Figure 11:**
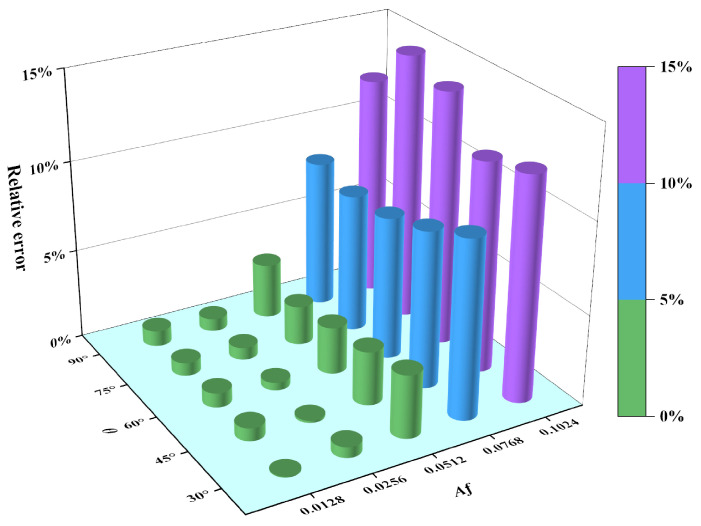
Distribution of relative errors between $K_{t\max}^{T_{1}}$ and ${K_{t\left|FEM\right.}^{T_{1}}}_{\max}$ for different cosine-wave surfaces.

Based on the data in Table 1, the relative errors between the maximum analytical SCFs $K_{t\max}^{T_{1}}$ and their counterparts ${K_{t\left|FEM\right.}^{T_{1}}}_{\max}$ were calculated, with the statistical distribution characteristic of these errors illustrated in Figure 11.

The results in Figure 11 show that, when Af< 0.05, the relative errors remain below 5%; however, when Af> 0.1, the relative errors significantly increase to over 10%. Further analysis reveals a statistically significant positive correlation between the relative error and Af. Notably, under constant-Af conditions, the influence of θ on the relative error demonstrates a non-monotonic characteristic.

### 4.3. The Influence of Poisson’s Ratio on SCFs

Based on Equation (23), four single-layer cosine-type 3D surfaces with a side length of 1 mm were constructed, featuring the following geometric parameters: surface amplitude A=0.01 mm, texture direction θ = 45°, and surface frequencies f= 1.28, 2.56, 5.12, and 7.68 ${\mathrm{mm}}^{-1}$, respectively. The expressions of the 3D surface morphologies are as follows:

(32) $$\begin{array}{ll} Surface\;\mathrm{IV}: & z\left(x,y\right)=0.01\cos\left[2\pi \cdot 1.28\cdot \left(x+y\right)\right]\\ Surface\;\;\;\mathrm{II}: & z\left(x,y\right)=0.01\cos\left[2\pi \cdot 2.56\cdot \left(x+y\right)\right]\\ Surface\;\;\;V: & z\left(x,y\right)=0.01\cos\left[2\pi \cdot 5.12\cdot \left(x+y\right)\right]\\ Surface\;\mathrm{VI}: & z\left(x,y\right)=0.01\cos\left[2\pi \cdot 7.68\cdot \left(x+y\right)\right]. \end{array}$$

By setting y=0.5 mm in Equation (32), the expressions for the centerline profile curves of the 3D surfaces are obtained as follows:

(33) $$\begin{array}{ll} Centerline\;\mathrm{IV}: & z\left(x\right)=0.01\cos\left[2\pi \cdot 1.28\cdot \left(x+0.5\right)\right]\\ Centerline\;\;\;\mathrm{II}: & z\left(x\right)=0.01\cos\left[2\pi \cdot 2.56\cdot \left(x+0.5\right)\right]\\ Centerline\;\;\;V: & z\left(x\right)=0.01\cos\left[2\pi \cdot 5.12\cdot \left(x+0.5\right)\right]\\ Centerline\;\mathrm{VI}: & z\left(x\right)=0.01\cos\left[2\pi \cdot 7.68\cdot \left(x+0.5\right)\right]. \end{array}$$

**Figure 12:**
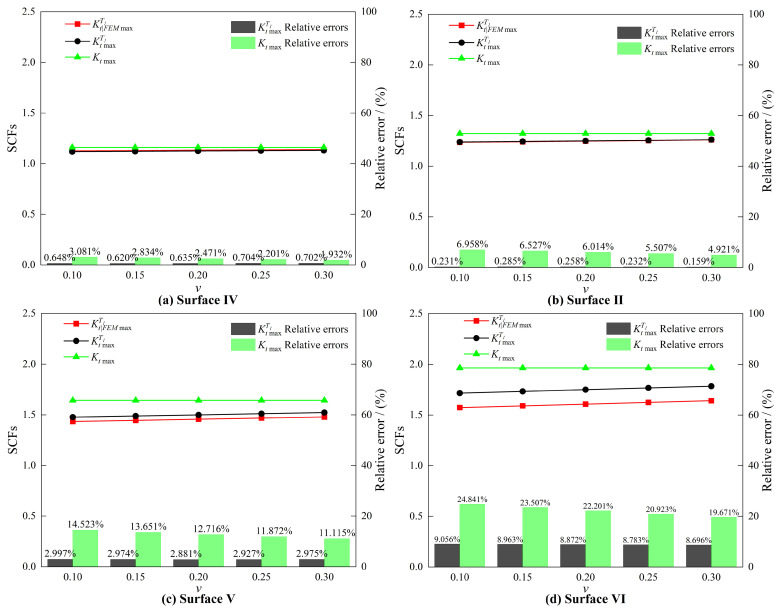
Comparisons of the maximum SCFs obtained by three methods for different surfaces under varying v.

A comparative analysis of stress concentration characteristics was conducted for v= 0.1, 0.15, 0.2, 0.25, and 0.3. Figure 12 illustrates the comparison of three key metrics under varying v: the maximum 3D analytical solution $K_{t\max}^{T_{1}}$ (Equation (27)), the maximum FEM solution ${K_{t\left|FEM\right.}^{T_{1}}}_{\max}$, and the maximum 2D analytical solution $K_{t\max}$ (Equation (29)), evaluated along the surface centerline far from the loading boundary.

The analysis results in Figure 12 reveal a high consistency in stress distributions between the 3D analytical solution and FEM simulations. Specifically, both the maximum 3D analytical SCF $K_{t\max}^{T_{1}}$ and the maximum FEM solution ${K_{t\left|FEM\right.}^{T_{1}}}_{\max}$ exhibit monotonically increasing trends with rising Poisson’s ratio v. In contrast, the maximum 2D analytical solution $K_{t\max}$ demonstrates no discernible correlation with variations in v.

With the gradual increase in surface frequency f, the relative error of the 2D analytical solution (Equation (29)) exhibits a significant rise. This phenomenon is primarily attributable to the dominant influence of the parameters A and f on SCFs within the 2D analytical framework, while critical factors such as Poisson’s ratio v and the texture direction parameter θ are neglected. In contrast, the 3D analytical solution (Equation (27)), incorporating both the material’s Poisson’s ratio and texture direction characteristics, demonstrates markedly improved predictive accuracy. Although its relative error also grows with increasing Af, the maximum relative error remains consistently below 10%, confirming the superior reliability and applicability of the 3D analytical solution for SCF prediction.

## 5. Application to Real Measured Surfaces

**Figure 13:**
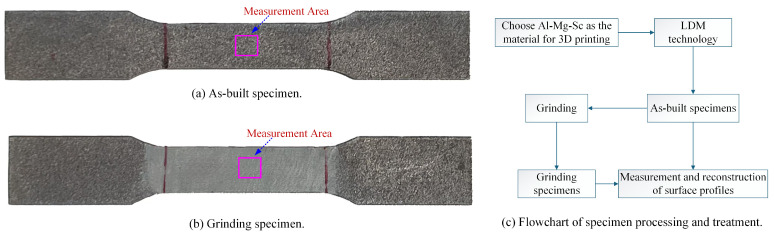
The 3D-printed experimental specimens and the flowchart.

In this section, we employed additive manufacturing (AM) technology to fabricate experimental specimens through the following procedure: Metallic powder materials were selected for the 3D printing process to produce as-built specimens with high roughness. Subsequently, the rough specimens were subjected to the grinding process to generate surface morphologies containing machining-induced texture features. Grinding reduced the surface roughness of the specimens while simultaneously imparting controlled surface textures tailored for the subsequent investigation, as shown in Figure 13, where Figure 13a displays the untreated, as-printed specimen retaining the characteristic layered morphology typical of AM processes, while Figure 13b presents the ground specimen with machining-induced texture features on its surface. Figure 13c schematically illustrates the flowchart of specimen processing and treatment.

**Figure 14:**
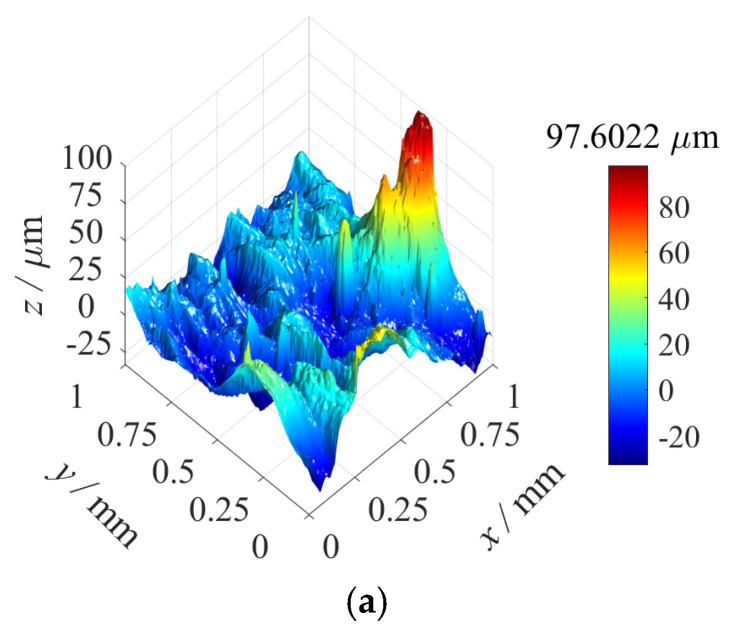

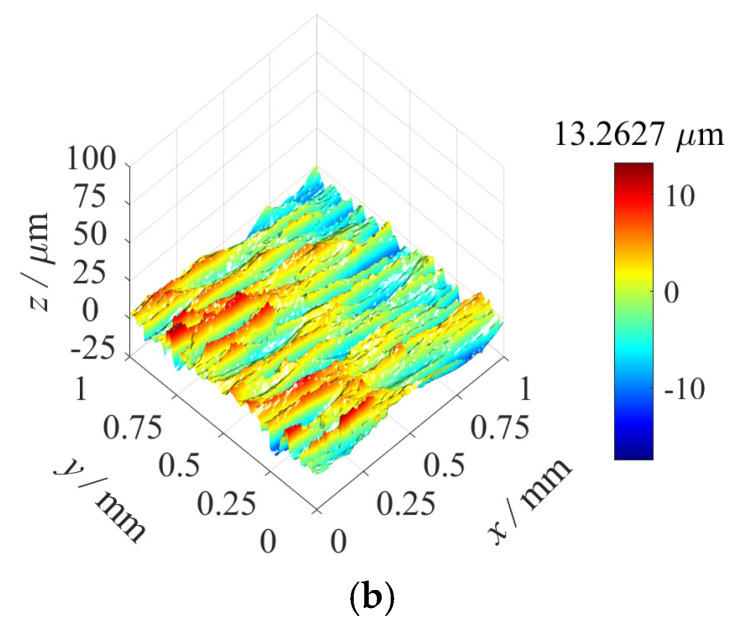
Surface morphologies of the 3D−printed specimens: (**a**) The AM as−built surface morphology. (**b**) Surface morphology after undergoing the grinding process.

The specimens’ surfaces were precisely measured using a white-light interferometer, with the measurement area selected at the central region to ensure an accurate representation of the overall surface characteristics and statistical significance. Figure 14a displays the untreated as-built surface morphology, while Figure 14b illustrates the post-grinding surface texture. During measurement, the white-light interferometer captured high-resolution topographic details and quantified the 3D surface roughness using the arithmetic mean height parameter $S_{a}=\frac{1}{MN}\sum_{i=1}^{M}\sum_{j=1}^{N}\left|Z_{ij}\right|$, where $Z_{ij}$ represents the height matrix over an M×N grid.

Specifically, the 3D machined surface morphology can be reconstructed by superimposing different frequency components using a 2D Fourier transform, as shown in Figure 1b. The formula for the 3D reconstructed surface morphology is expressed as follows:

(34) $$z\left(x,y\right)=-\sum_{i=1}^{M}\sum_{j=1}^{N}A_{ij}\cos\left(2\pi \left(f_{xi}x+f_{yj}y\right)+\varphi_{ij}\right),$$

where $A_{ij}$, $f_{xi}$, $f_{yj}$, and $\varphi_{ij}$ represent the amplitude, frequencies in the *x* and *y* directions, and phase of the surface wave components, respectively.

Combining Equations (34) and (6), the normal SCFs of the 3D reconstructed surface topography under uniaxial tensile loading can be derived as follows:

(35) $$\begin{array}{l} K_{t}^{T_{1}}(x,y)=1+4\pi \sum_{i=1}^{M}\sum_{j=1}^{N}A_{ij}\;\;\;\;\\ \;\;\;\;\times \frac{f_{xi}^{4}+(1+v)f_{xi}^{2}f_{yj}^{2}}{\left(f_{xi}^{2}+f_{yj}^{2}\right)\sqrt{f_{xi}^{2}+f_{yj}^{2}}}\cos\left(2\pi \left(f_{xi}x+f_{yj}y\right)+\varphi_{ij}\right). \end{array}$$

For the centerline profile extracted from the 3D surface topography, Fourier harmonic reconstruction can also be performed, as illustrated in Figure 1c. The reconstructed centerline profile and its corresponding SCF calculation formula are presented below [47]:

(36) $$z\left(x\right)=-\sum_{i=1}^{n}A_{i}\cos\left(2\pi f_{i}x+\varphi_{i}\right).$$

(37) $$K_{t}=1+4\pi \sum_{i=1}^{n}A_{i}f_{i}\cos\left(2\pi f_{i}x+\varphi_{i}\right).$$

It should be noted that, for arbitrary surfaces with slight undulations, the slope parameter along the surface should be far less than 1. For a 3D surface represented by Fourier series, the condition $4\pi^{2}\sum_{i=1}^{M}\sum_{j=1}^{N}\left(f_{xi}^{2}+f_{yj}^{2}\right)A_{ij}^{2}\ll 1$ needs to be satisfied. In comparison, for its 2D counterpart, the requirement is $2\pi^{2}\sum_{i=1}^{n}A_{i}^{2}f_{i}^{2}\ll 1$.

The inherently high surface roughness of AM components typically induces significant stress concentration phenomena, which critically impair their fatigue performance and service life. Consequently, surface treatment processes such as grinding are routinely applied to AM parts in engineering practice to enhance their surface integrity. This study directly analyzes the post-grinding specimen surface, thereby more accurately reflecting actual engineering surface conditions and their effects on stress concentration behaviors.

During surface reconstruction, once the surface undulations maintain microscale magnitudes, the SCFs can be calculated via Equation (35), regardless of the number of frequency components employed. Cheng et al. demonstrated that high-frequency components exert minimal impact on structural fatigue behavior [44]. Setting a high frequency cut-off for surface reconstruction is therefore justified.

**Figure 15:**
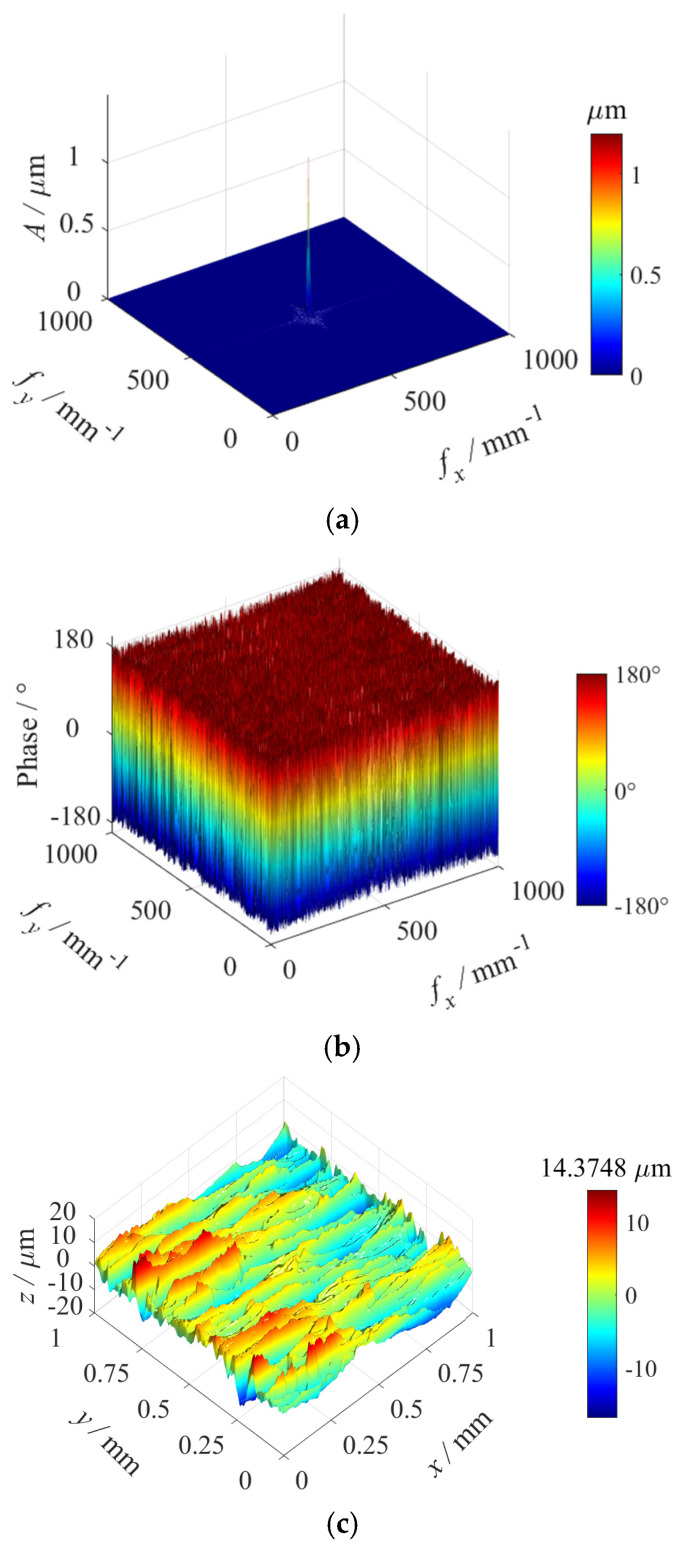
Amplitude spectrum, phase spectrum, and the reconstructed surface morphology: (**a**) Amplitude spectrum of the original surface morphology. (**b**) Phase spectrum of the original surface morphology. (**c**) The 3D reconstructed surface morphology.

The original surface morphology (as shown in Figure 14b) was subjected to spectral analysis through the 2D Fourier transform, yielding the corresponding amplitude and phase spectra (as shown in Figure 15a,b). Based on the results of the spectral analysis, approximately 80 surface harmonic components were employed from 3D surface reconstruction, and the reconstructed surface morphology is shown in Figure 15c. The arithmetic mean height $S_{a}$ of the original surface topography was 3.52 μm, and the $S_{a}$ of the reconstructed surface topography was 3.51 μm.

**Figure 16:**
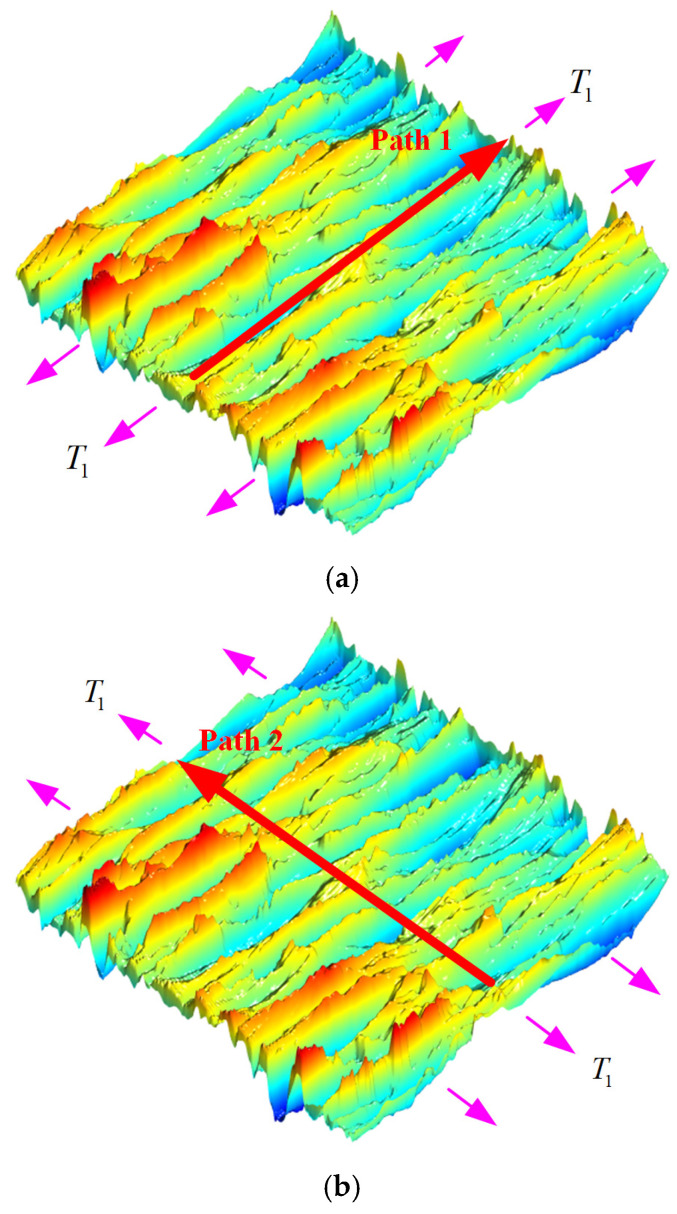
Schematic of surface texture direction and loading direction: (**a**) Texture direction approximately parallel to the loading direction. (**b**) Texture direction approximately perpendicular to the loading direction.

As shown in Figure 16a, the surface texture direction is approximately parallel to the loading direction $T_{1}$. To systematically investigate the influence of the texture direction on stress concentration effects, this study established a theoretical comparative scenario where the texture direction was approximately perpendicular to the $T_{1}$ direction (Figure 16b). Comparative analysis of the surface stress distributions under these two configurations effectively reveals the governing patterns of the texture direction on SCFs.

Experimental determination of the SCFs for 3D surface morphologies presents significant challenges. The FEM has become the preferred approach for verifying the effectiveness of the analytical solutions of SCFs in AM surface morphologies. In this section, the FEM of the 3D reconstructed surface topography is established (refer to Section 4.1 for the modeling procedures) to verify the accuracy of the theoretical predictions.

**Figure 17:**
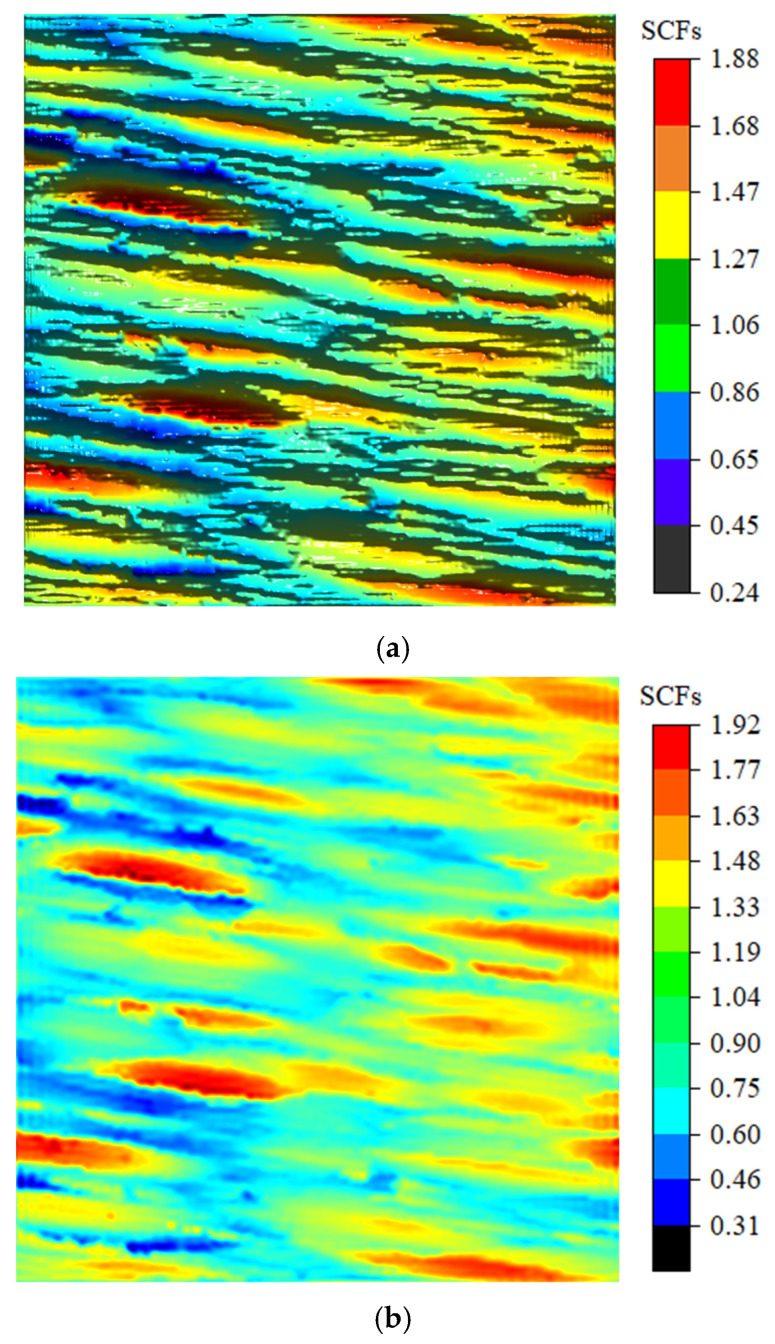
Comparison between analytical solutions and FEM simulation results for SCFs under v=0.3, with $T_{1}$ approximately parallel to the texture direction: (**a**) The 3D analytical solution $K_{t}^{T_{1}}$ for v=0.3. (**b**) FEM results $K_{t\left|FEM\right.}^{T_{1}}$ for v=0.3.

**Figure 18:**
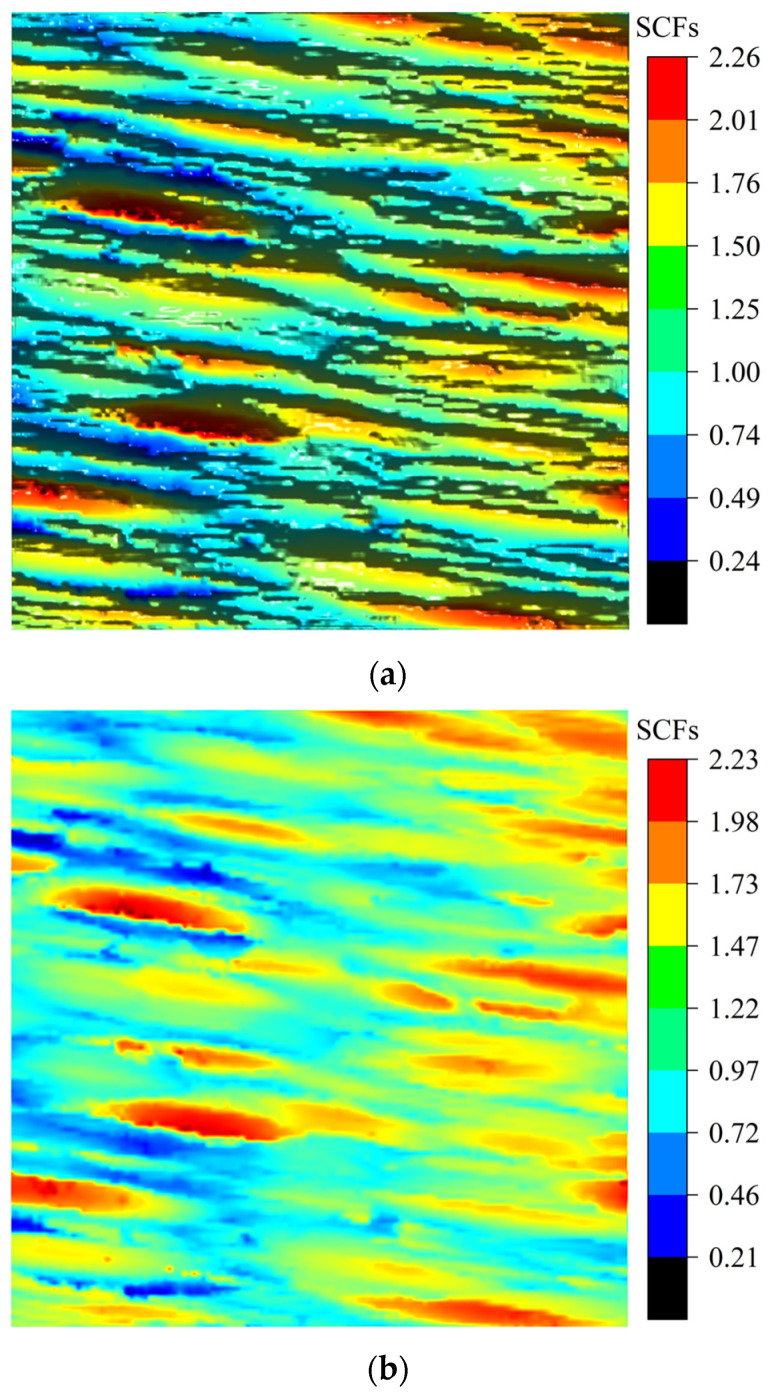
Comparison between analytical solutions and FEM simulation results for SCFs under v=0.3, with $T_{1}$ approximately perpendicular to the texture direction: (**a**) The 3D analytical solution $K_{t}^{T_{1}}$ for v=0.3. (**b**) FEM results $K_{t\left|FEM\right.}^{T_{1}}$ for v=0.3.

Under the condition of v=0.3 with the loading direction $T_{1}$ approximately parallel to the texture direction, the analytical solution for normal SCFs along the $T_{1}$ direction, derived from Equation (35), is as shown in Figure 17a, while Figure 17b presents the corresponding FEM simulation results. When the $T_{1}$ direction is approximately perpendicular to the texture direction, the analytical solution for normal SCFs is as illustrated in Figure 18a, with the FEM results provided in Figure 18b.

**Figure 19:**
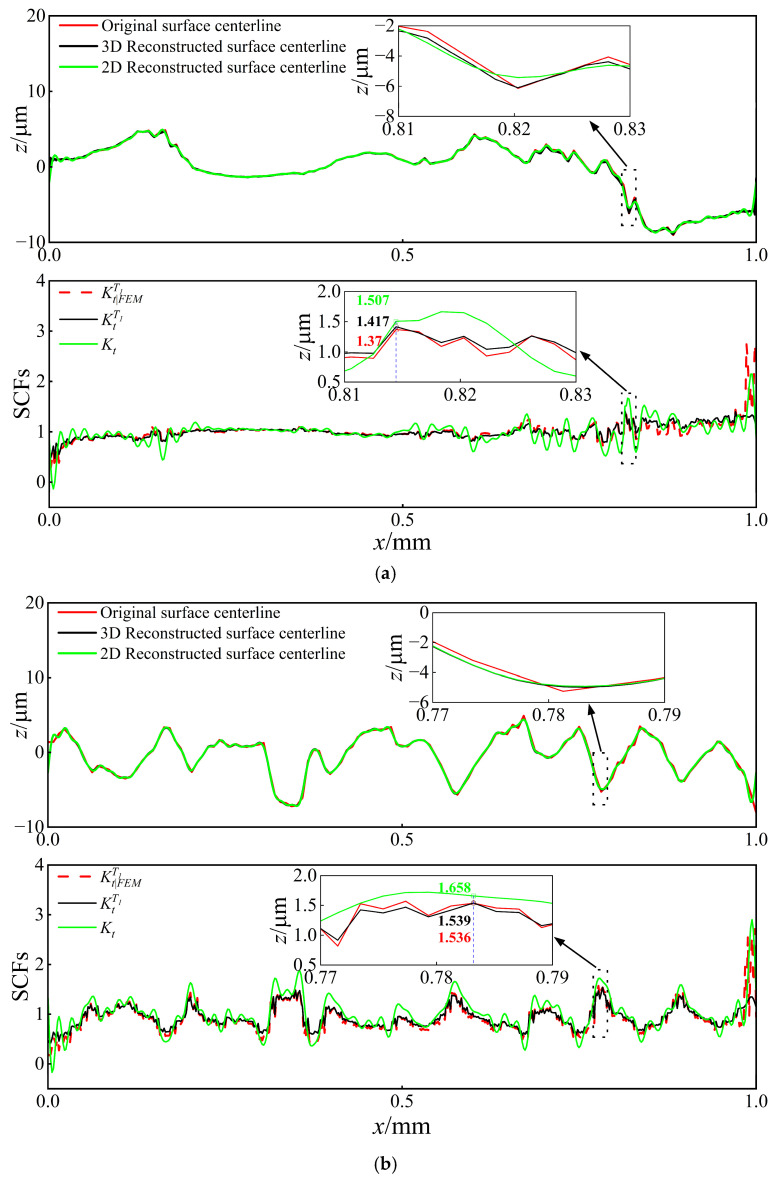
Comparison of theoretical and FEM results for SCFs along Path 1 and Path 2 with v=0.3: (**a**) Comparison of $K_{t}$, $K_{t}^{T_{1}}$, and $K_{t\left|FEM\right.}^{T_{1}}$ along Path 1. (**b**) Comparison of $K_{t}$, $K_{t}^{T_{1}}$, and $K_{t\left|FEM\right.}^{T_{1}}$ along Path 2.

To better characterize the relationship between SCFs and surface morphology, the surface centerline profile was extracted for analysis. Fourier transform was applied to the original centerline profile to reconstruct the surface contour and derive the 2D analytical solution $K_{t}$ (Equation (37)). A 2D Fourier transform was carried out on the original 3D surface to generate the 3D reconstructed morphology, from which the centerline profile was extracted to obtain the 3D analytical solution $K_{t}^{T_{1}}$ (Equation (35)). Figure 19 presents a comparative analysis of $K_{t}$, $K_{t}^{T_{1}}$, and the FEM simulation results $K_{t\left|FEM\right.}^{T_{1}}$ along the centerline profile (Path 1 and Path 2).

Figure 19 shows that the stress increases at the wave valleys and decreases at each peak. Due to the existence of stress singularity at the FEM boundaries, the analysis primarily focuses on the stress concentration away from the loading boundary, where the 3D analytical solutions demonstrate close agreement with the FEM simulation results; however, the error of the 2D analytical solution appears relatively large.

As shown in Figure 19, the maximum SCFs $K_{t\max}^{T_{1}}$ along Path 1 and Path 2 are 1.42 and 1.54, respectively, indicating an 8.5% increase in stress concentration level under the perpendicular loading condition compared to the parallel loading. Comparative analysis of Figure 18 and Figure 19 reveals that when the loading direction is parallel or perpendicular to the texture direction, the maximum FEM results ${K_{t\left|FEM\right.}^{T_{1}}}_{\max}$ are 1.92 and 2.23, respectively, while the maximum 3D analytical solutions $K_{t\max}^{T_{1}}$ yield 1.88 and 2.26, respectively. These results demonstrate a significant elevation of SCFs under perpendicular loading conditions, with FEM and analytical solutions showing increases of 16.1% and 20.2%, respectively.

The comparative analysis confirms strong consistency between FEM simulations and theoretical solutions: Under practical engineering conditions, SCFs exhibit a positive correlation with the texture direction parameter θ, i.e., the SCFs increase as θ grows. This pattern highlights the machining texture direction as a critical factor influencing stress concentration behavior, providing essential theoretical guidance for optimizing surface texture design in engineering applications. This finding is consistent with the statements reported by Chen et al. [49].

Table 2 lists the SCFs analysis results at locations away from the loading boundary along Path 1, under the condition that the texture direction is parallel to the loading direction, including the maximum FEM simulation value ${K_{t\left|FEM\right.}^{T_{1}}}_{\max}$, 3D analytical solution $K_{t\max}^{T_{1}}$, 2D analytical solution $K_{t\max}$, and the relative errors between $K_{t\max}$, $K_{t\max}^{T_{1}}$, and ${K_{t\left|FEM\right.}^{T_{1}}}_{\max}$.

Analysis of the data from Table 2 demonstrates that the 3D analytical solution achieves higher computational accuracy than its 2D counterpart, with significantly reduced relative errors. Specifically, the 3D analytical solution accurately captures the increasing trend of SCFs with rising Poisson’s ratio v, whereas the 2D solution yields constant values independent of v. This comparative analysis confirms that Poisson’s ratio v is a critical parameter influencing stress concentration behavior in practical engineering scenarios. Consequently, the 3D analytical method proves more suitable for analyzing and predicting stress concentration problems under practical engineering conditions, compared to traditional 2D approaches.

## 6. Conclusions

This study establishes a general 3D analytical framework for the SCFs induced by micro-undulated surfaces based on the boundary perturbation method, with systematic analyses revealing the regulatory mechanism of surfaces with different geometric features on stress concentration effects. The effects of Poisson’s ratio and the surface texture direction are incorporated into the analytical formulae as well. The key findings can be summarized as follows:

(1) Influence of Poisson’s ratio: The proposed 3D analytical solution distinctly reveals a positive correlation between v and SCFs. This correlation represents a significant finding that conventional 2D models fail to capture.
(2) Influence of texture directionality: Non-orthogonal surface textures exhibit remarkable anisotropic stress modulation, which is governed by the texture directional parameter θ. The 3D analytical solution quantitatively captures the phenomenon where SCFs increase monotonically with θ. In contrast, 2D solutions are unable to account for this directional sensitivity. A parametric analysis of single-layer cosine-wave surfaces further confirms a nonlinear relationship between the amplitude–frequency product Af and theoretical accuracy.
(3) Validation with real surface morphologies: Application to machined surfaces demonstrates the superior predictive capability of the 3D solution, achieving an average of 10% higher accuracy in SCF prediction compared to 2D methods, with relative errors stably maintained within 5%. The analysis further verifies that SCFs increase with increasing v and are highly sensitive to surface texture directionality.

Future work: To further broaden the applicability of this theoretical framework, further research will focus on extending it to analyze surface SCFs in anisotropic materials subjected to uniaxial or multiaxial loading. Additionally, applications to surface evolution processes, including surface diffusion and chemical etching, will be explored. Comparative studies involving experimental data or alternative numerical models, such as phase-field or crystal plasticity simulations, could refine the predictive capabilities of the framework.

## Figures and Tables

**Table 1:** Maximum values of SCFs from FEM simulations and 3D analytical solutions for different cosine-wave surfaces.

Af		θ				
		30°	45°	60°	75°	90°
0.0128	${K_{t\left|FEM\right.}^{T_{1}}}_{\max}$	1.098	1.139	1.159	1.167	1.171
	$K_{t\max}^{T_{1}}$	1.099	1.131	1.15	1.584	1.161
0.0256	${K_{t\left|FEM\right.}^{T_{1}}}_{\max}$	1.190	1.260	1.294	1.326	1.331
	$K_{t\max}^{T_{1}}$	1.197	1.262	1.299	1.317	1.322
0.0512	${K_{t\left|FEM\right.}^{T_{1}}}_{\max}$	1.347	1.479	1.558	1.599	1.695
	$K_{t\max}^{T_{1}}$	1.394	1.523	1.599	1.634	1.643
0.0768	${K_{t\left|FEM\right.}^{T_{1}}}_{\max}$	1.439	1.642	1.759	1.811	1.816
	$K_{t\max}^{T_{1}}$	1.591	1.785	1.899	1.951	1.965
0.1024	${K_{t\left|FEM\right.}^{T_{1}}}_{\max}$	1.592	1.833	1.927	1.975	2.037
	$K_{t\max}^{T_{1}}$	1.788	2.046	2.198	2.268	2.287

**Table 2:** Relative errors of maximum normal SCFs between analytical solutions and FEM results at locations away from the loading boundary along Path 1.

v	${K_{t\left|FEM\right.}^{T_{1}}}_{\max}$	$K_{t\max}^{T_{1}}$ by Equation (35)	$K_{t\max}$ by Equation (37)	Relative Errors of 3D Analytical Solution	Relative Errors of 2D Analytical Solution
0.1	1.350	1.386	1.507	2.64%	11.63%
0.15	1.355	1.390		2.55%	11.22%
0.2	1.359	1.392		2.45%	10.89%
0.25	1.365	1.397		2.34%	10.40%
0.3	1.370	1.417		3.44%	10.00%

## Data Availability

The original contributions presented in the study are included in the article, further inquiries can be directed to the corresponding author.

## Nomenclature

a	Notch depth of semi-ellipse notch
A/ε	Amplitude for sinusoidal and single-layer cosine wave/Gaussian notch
$A_{i}$/$A_{ij}$	Amplitudes of the surface wave components in 2D/3D case
b	Notch half-width of semi-ellipse notch
C	Loading ratio
f	Surface frequency for single-layer cosine wave
$f_{x}/f_{y}$	Surface frequency along x/y direction
$f_{i}$/$f_{xi},\;f_{yj}$	Frequencies of the surface wave components in 2D/3D case
$\overset{\wedge }{G_{xx}},\;\overset{\wedge }{G_{yy}},\overset{\wedge }{\;G_{xy}}$	Kernel functions
$K_{t}$	Analytical solution of normal SCFs in 2D case
$K_{t}^{T_{1}},\;K_{t}^{T_{2}}$	Analytical solution of normal SCFs in 3D case
$K_{t\left|FEM\right.}^{T_{1}}$	SCFs from finite element simulation results
$K_{t\max}$	Maximum value of the analytical solution for 2D SCFs
$K_{t\max}^{T_{1}}$	Maximum value of the analytical solution for 3D SCFs
${K_{t\left|FEM\right.}^{T_{1}}}_{\max}$	Maximum SCFs from finite element simulation results
$K_{ts}$	Analytical solution of shear SCFs in 3D case
R	The maximum stress perturbation ratio
r	Distance between two points
$S_{a}$	The arithmetic mean height parameter
$T_{i}$	Loading
v	Poisson’s ratio
$z\left(x\right)$	Two-dimensional surface profile
$z\left(x,y\right)$	Three-dimensional surface topography
$Z_{ij}$	Height matrix
σ	The standard deviation
$u_{i},\;\sigma_{ij}$	Displacement and stress
$\varphi_{i}$/$\varphi_{ij}$	Phases of the surface wave components in 2D/3D case
λ	Wavelength parameter
β	The coefficient
θ	Texture direction parameter
